# Type 2 diabetes mellitus is associated with a lower fibrous cap thickness but has no impact on calcification morphology: an intracoronary optical coherence tomography study

**DOI:** 10.1186/s12933-017-0635-2

**Published:** 2017-12-01

**Authors:** Andrea Milzi, Mathias Burgmaier, Kathrin Burgmaier, Martin Hellmich, Nikolaus Marx, Sebastian Reith

**Affiliations:** 1 Department of Internal Medicine I–Cardiology, RWTH Aachen University, Pauwelsstr. 30, 52074 Aachen, Germany; 2 Department of Pediatrics, University Hospital of Cologne, Cologne, Germany; 3 Institute of Medical Statistics and Computational Biology, University of Cologne, Cologne, Germany

**Keywords:** Coronary calcification, Optical coherence tomography, Diabetes mellitus, Plaque vulnerability

## Abstract

**Background:**

Patients with type 2 diabetes (T2DM) are at high risk for cardiovascular events, which usually arise from the rupture of a vulnerable coronary plaque. The minimal fibrous cap thickness (FCT) overlying a necrotic lipid core is an established predictor for plaque rupture. Recently, coronary calcification has emerged as a relevant feature of plaque vulnerability. However, the impact of T2DM on these morphological plaque parameters is largely unexplored. Therefore, this study aimed to compare differences of coronary plaque morphology in patients with and without T2DM with a particular focus on coronary calcification.

**Methods:**

In 91 patients (T2DM = 56, non-T2DM = 35) with 105 coronary de novo lesions (T2DM = 56, non-T2DM = 49) plaque morphology and calcification were analyzed using optical coherence tomography (OCT) prior to coronary intervention.

**Results:**

Patients with T2DM had a lower minimal FCT (80.4 ± 27.0 µm vs. 106.8 ± 27.8 µm, p < 0.001) and a higher percent area stenosis (77.9 ± 8.1% vs. 71.7 ± 11.2%, p = 0.001) compared to non-diabetic subjects. However, patients with and without T2DM had a similar total number of calcifications (4.0 ± 2.6 vs. 4.2 ± 3.1, p = ns) and no significant difference was detected in the number of micro- (0.34 ± 0.79 vs. 0.31 ± 0.71), spotty (2.11 ± 1.77 vs. 2.37 ± 1.89) or macro-calcifications (1.55 ± 1.13 vs. 1.53 ± 0.71, all p = ns). The mean calcium arc (82.3 ± 44.8° vs. 73.7 ± 31.6), the mean thickness of calcification (0.54 ± 0.13 mm vs. 0.51 ± 0.15 mm), the mean calcified area (0.99 ± 0.72 mm^2^ vs. 0.78 ± 0.49 mm^2^), the mean depth of calcification (172 ± 192 μm vs. 160 ± 76 μm) and the cap thickness overlying the calcification (50 ± 71 μm vs. 62 ± 61 μm) did not differ between the diabetic and non-diabetic groups (all p = ns).

**Conclusion:**

T2DM has an impact on the minimal FCT of the coronary target lesion, but not on localization, size, shape or extent of calcification. Thus, the minimal FCT overlying the necrotic lipid core but not calcification is likely to contribute to the increased plaque vulnerability observed in patients with T2DM.

## Background

Type 2 diabetes mellitus (T2DM) is a recognized risk factor for coronary atherosclerosis and subsequent cardiovascular events which results in a two- to threefold higher mortality risk compared to patients without T2DM [1, 2]. Several investigations have indicated that T2DM patients without a history of cardiovascular events have a similar chance for myocardial infarction as compared to non-T2DM patients with previous coronary events [2, 3]. Cardiovascular events usually arise from rupture of a vulnerable, coronary plaque [4]. We and others have demonstrated using optical coherence tomography (OCT), that coronary lesions of patients with T2DM are characterized by several features of plaque vulnerability, including a higher frequency of thin-capped fibroatheromas (TCFA), a larger lipid core, the presence of microvessels and/or macrophages suggesting coronary inflammation [5, 6].

Recently, coronary calcification has been discussed as an additional risk factor of coronary plaque vulnerability. Specifically, investigations using computed tomography and intravascular ultrasound (IVUS) have indicated that the extent of coronary calcification generally correlates with atherosclerotic plaque burden as well as with the incidence of adverse cardiac events [7]. Furthermore, superficial microcalcifications may be responsible for a higher tissue stress [8, 9] and thus may induce a higher rate of cardiovascular events [9–11]. Moreover, calcified nodules, defined as calcifications protruding into the lumen with an overlying fibrous cap disruption, may be a novel morphological feature causing an acute coronary syndrome (ACS) [12]. Taken together, the contribution of plaque calcification to plaque vulnerability appears to be dependent on presence, size and localization of calcifications within lesions. To date, the impact of T2DM on these parameters is unknown. Due to its superior resolution of approximately 10–20 µm, OCT allows a supreme “in vivo” visualization of different plaque morphologies as well as a precise quantification of coronary luminal and intraluminal parameters [13]. Given that particularly small and superficial calcifications contribute to lesion vulnerability, OCT presently represents the most accurate method to determine localization, size, shape and extent of calcifications within coronary lesions in vivo [14].

Due to the enhanced cardiovascular event rate of patients with T2DM, the aim of this study was to compare differences of coronary plaque morphology in patients with and without T2DM by OCT. As micro-calcifications are a novel feature of coronary plaque vulnerability, this study investigated if the increased cardiovascular risk of patients with T2DM may be explained by an altered localization, size, shape or extent of calcifications within coronary lesions.

## Methods

### Study population

**Fig. 1:**
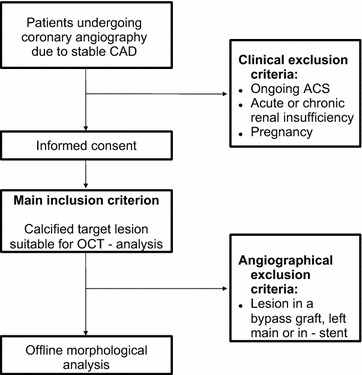
Study design algorithm including inclusion and exclusion criteria is depicted. ACS, acute coronary syndrome; CAD, coronary artery disease; OCT, optical coherence tomography

In this study, 98 patients with stable coronary artery disease (CAD) and 112 de novo coronary target lesions were examined with coronary angiography and coronary OCT-analysis prior to coronary intervention at the Department of Cardiology of the University Hospital of the RWTH Aachen between July 2014 and January 2017. All patients underwent coronary angiography due to stable CAD. Main inclusion criterion was the presence of a coronary stenosis suitable for OCT analyses, in which the OCT examination itself demonstrated the presence of calcification. Of the initial 98 patients with 112 lesions included in the study, 7 patients with 7 lesions were excluded due to the absence of calcification in the OCT-analysis, resulting in 105 lesions from 91 patients. The study cohort was divided into a diabetic (56 patients, 56 lesions) and non-diabetic group (35 patients, 49 lesions, Fig. 1). This number of patients has previously been sufficient to show differences in OCT-derived plaque morphology including plaque calcification between patients with and without diabetes [15]. The diagnosis of T2DM was based on clinical history, ongoing antidiabetic therapy and/or an Hb_A1C_ > 6.5%. Stable CAD at the time of intervention was defined as no progression of severity, frequency and duration of clinical symptoms within the previous 6 weeks. Target lesion identification was based on the coronary angiogram with an at least 40% stenosis suitable for coronary intervention and confirmed by the combination of echocardiographic wall motion abnormalities, positive stress testing in MRT or echocardiography and/or evidence of hemodynamic relevance of the coronary lesion as assessed by fractional flow reserve (FFR) measurements. The presence of significant myocardial ischemia and not plaque morphology/vulnerability was used to guide coronary intervention.

Exclusion criteria were left main coronary artery stenosis, bifurcation and bypass graft lesions, in-stent restenosis, an ongoing ACS, an acute or chronic renal insufficiency and pregnancy. Written informed consent of all patients was obtained. The study was approved by the local ethics committee and is in accordance with the declaration of Helsinki on ethical principles for medical research involving human subjects.

### Image acquisition

The OCT images in the target lesions were acquired using a Frequency-Domain-OCT C7XR system and the DragonFly catheter (St. Jude Medical Systems; Lightlab Imaging, Inc, Westford, Massachusetts, USA). Complete blood removal as a prerequisite for image acquisition using the infrared light technique of OCT was obtained by the injection of 14 ml contrast (iodixanol) at a flow rate of 4 ml/s through the guiding catheter. The image acquisition was obtained with an automated pull-back at a rate of 20 mm/s. The following offline plaque analysis was performed by two independent observers with an extensive experience in OCT imaging analysis[16–20], blinded to the study protocol. The analysis was carried out throughout the entire lesion frame by frame in 0.4 mm intervals using St. Jude’s proprietary software. In case of discordant results a consensus measurement was taken. The intraclass correlation coefficients for intra- and interobserver agreements were 0.979 and 0.893 for calcium arc, 0.989 and 0.902 for calcium area and 0.949 and 0.927 for percent area stenosis.

### Morphological analysis

The OCT image analysis was performed over the entire segment of the pull-back image (54 mm). The morphological and quantitative intraluminal analysis of the plaque was performed as previously described by Tearney et al. [21]. The presence of calcified (signal-poor heterogeneous region with clearly delineated contours) and lipid (signal-poor homogeneous region without borders and high signal attenuation) regions were assessed. Two calcifications were considered as parted when they were longitudinally separated by at least 1 mm or when they were detectable on different portions of the single slice image without any contact or continuity throughout the whole length of the calcifications themselves.

**Fig. 2:**
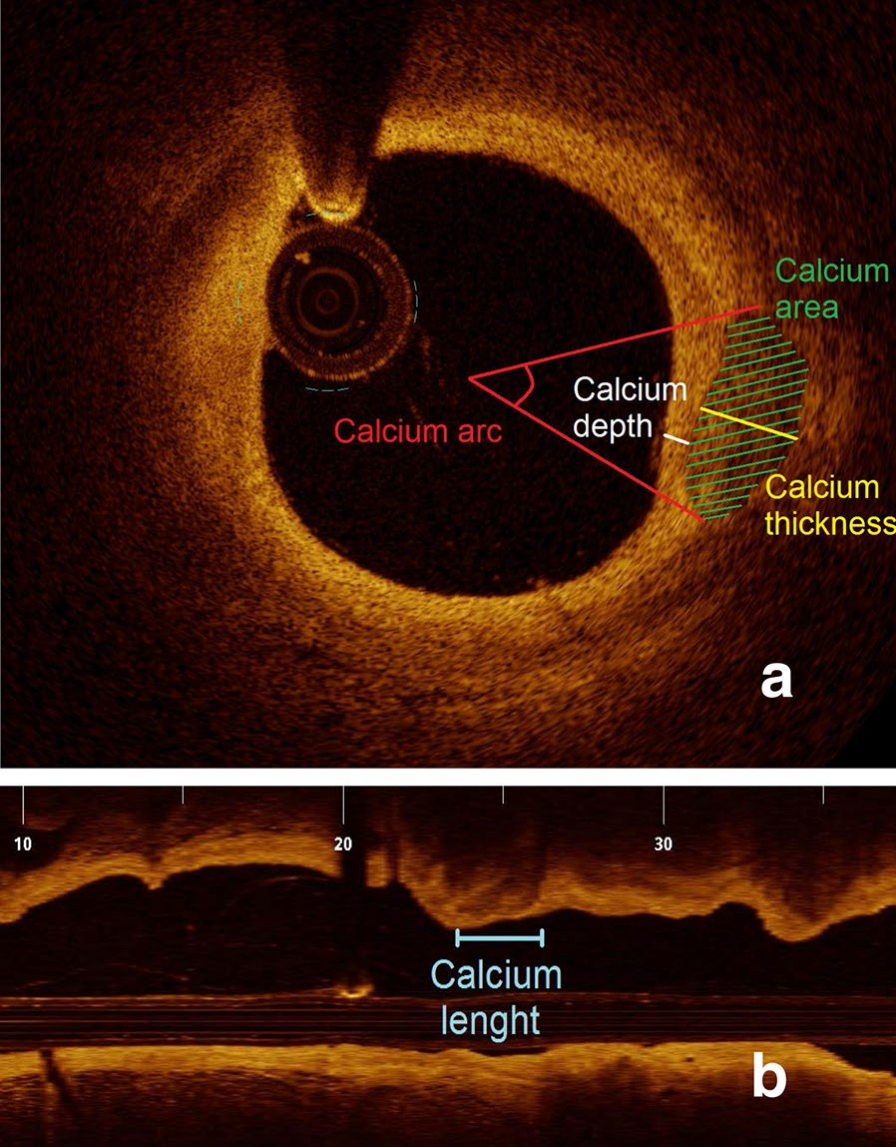
Details of calcification analysis in short (**a**) and long axis (**b**). Quantitative analysis of various parameters for the assessment of localization, size, shape and extent of calcifications in a transverse (**a**) and longitudinal (**b**) section of an OCT—image of a fibrocalcific coronary plaque

Analysis of each calcified region by OCT consisted of the measurement of the following parameters and as displayed in Fig. 2:

- Calcium arc, defined as the widest angle in which the calcifications were detectable;
- calcium depth, defined as the distance between the most superficial edge of calcification and the vessel lumen;
- calcium thickness, defined as the maximal diameter of the calcification, measured perpendicularly to the lumen/intima interface;
- calcium area, measured manually following the sharply delineated contour of each calcium deposit. In case the contour was partly not detectable, the automatic interpolation function of the analytic software was used;
- calcium length, defined as the total length of calcifications in the whole plaque;
- calcium index, defined as the product of the mean calcium arc and the total calcium length, as previously described by Ong et al. [22].

In analogy to previous intravascular imaging studies assessing coronary calcifications [23–25], each plaque was furthermore characterized and classified according to the maximal calcium angle as:

- Spotty calcification, defined as a calcium length < 4 mm and a maximal calcium angle ranging from 22.5° to 90°;
- macrocalcification, characterized by a maximal calcium angle > 90°;
- microcalcification, in case of a maximal calcium angle < 22.5° and a maximal calcification length < 1 mm.

Due to the absence of established OCT criteria defining superficial calcifications and in analogy to a previous study from Ong [22], we defined single deposits as superficial-65 and superficial-100 according to their minimal calcium depth ≤ 65 or ≤ 100 µm, respectively.

### Statistical analysis

All statistical analyses were performed with SPSS software (IBM Corp., Armonk, NY, USA). Binary variables were summarized as count (percentage), continuous variables as mean ± standard deviation. The data were analyzed both on a per-patient basis for clinical characteristics and on a per-stenosis basis for lesion morphology. The statistical test did not account for the correlation of multiple plaques within patients. Distributions of continuous variables were compared with *t* test. Binary variables were compared by Pearson’s Chi squared test.

To account for possibly confounding differences between patients in localization, size, shape or extent of calcifications within coronary lesions with and without diabetes, covariate adjusting to the propensity score was performed. The propensity score was calculated using a multivariable binary logistic regression analysis with sex, age, mean arterial pressure, body mass index, total pack years and LDL-cholesterol as covariates. Covariate adjusting to the propensity score was performed using multivariable binary logistic regression analysis for binary variables or multivariable linear regression analysis for continuous variables. Statistical significance was awarded for p < 0.05.

## Results

### Study population

The 105 analyzed calcified lesions from 91 patients were categorized into two groups of 56 lesions from 56 patients with T2DM and 49 lesions from 35 patients without T2DM. Regarding cardiovascular risk factors, patients with T2DM showed a significantly higher body mass index (30.87 ± 4.58 kg/m^2^ vs. 27.75 ± 4.25 kg/m^2^, p < 0.001) and a higher rate of hyperlipidemia (69.65% vs. 45.71%, p = 0.04), whereas patients without T2DM presented with a higher cumulative tobacco exposure (32.71 ± 34.39 vs. 17.09 ± 22.19 pack years, p < 0.001). However, the quantitative number of active smokers at the time of study inclusion did not differ significantly (25.71% vs. 16.07%, p = 0.26). Further clinical characteristics of the two groups are reported in Table 1.

**Table 1:** Population analysis

	Non DM (35)	DM (56)	p value
Sex (male, %)	28 (80.0)	36 (64.3)	NS
Age (years)	70.7 ± 9.4	70.4 ± 5.9	NS
MAP (mmHg)	97 ± 12	96 ± 12	NS
BMI (kg/m^2^)	27.7 ± 4.2	30.9 ± 4.6	< 0.001
Positive family history (n, %)	17 (48.6)	25 (44.6)	NS
History of hypertension (n, %)	30 (85.7)	49 (87.5)	NS
Hyperlipidaemia (n, %)	16 (45.7)	39 (69.6)	0.04
Current smoking (n, %)	9 (25.7)	9 (16.1)	NS
Total pack years	32.7 ± 34.4	17.1 ± 22.2	< 0.001
COPD (n, %)	3 (8.6)	6 (10.7)	NS
Known CAD (n, %)	12 (34.3)	20 (35.7)	NS
Previous PCI (n, %)	11 (31.4)	14 (25.0)	NS
Previous CABG (n, %)	1 (2.9)	2 (3.6)	NS
Angina (CCS class)	1.9 ± 1.1	2.3 ± 0.7	NS
Dyspnoe (NYHA class)	1.6 ± 1.1	2.1 ± 0.8	NS
Duration of diabetes (years)	NA	11.9 ± 9.4	NA
Diabetic retinopathy (n, %)	NA	10 (17.9)	NA
Diabetic polyneuropathy (n, %)	NA	20 (35.7)	NA
Total cholesterol (mg/dl)	195.3 ± 43.8	191.2 ± 45.1	NS
LDL-cholesterol (mg/dl)	128.9 ± 39.6	117.8 ± 36.4	NS
HDL-cholesterol (mg/dl)	48 ± 13.2	44.2 ± 10.3	NS
HbA1c (%)	5.7 ± 0.4	7.2 ± 1.6	< 0.001
Medication prior to coronary angiography			
ASA (n, %)	31 (88.6)	52 (92.9)	NS
ACEi/ARB (n, %)	24 (65.7)	40 (71.4)	NS
β-Blocker (n, %)	24 (65.7)	46 (82.1)	NS
Statine (n, %)	21 (60.0)	35 (62.5)	NS

The data are presented as mean ± SD or n (%) and displayed with p values

DM, diabetes mellitus; BP, blood pressure; MAP, mean arterial pressure; BMI, body mass index; COPD, chronic obstructive pulmonary disease; CAD, coronary artery disease; PCI, percutaneous coronary intervention; CABG, coronary artery bypass graft; CCS, Canadian Cardiovascular Society; NYHA, New York Heart Association; LDL, low density lipoprotein, HDL, high density cholesterol; ASA, acetylsalicylic-acid; ACEi/ARB, angiotensin converting enzyme inhibitors/angiotensin receptor blockers; NS, not significant; NA, not applicable

### Extent and morphology of calcification

In both, patients with and without T2DM, a similar amount of calcifications within the coronary target vessel was detected. Similarly, the amount of micro- (0.34 ± 0.79 vs. 0.31 ± 0.71), spotty (2.11 ± 1.77 vs. 2.37 ± 1.89) or macro-calcifications (1.55 ± 1.13 vs. 1.53 ± 0.71) did not differ significantly between the diabetic and non-diabetic group. However, a significantly higher frequency of spotty calcifications could be found in patients without T2DM (93.90%) compared to those with T2DM (78.57%, p = 0.025). Furthermore, no significant difference was found in the measurements of the remaining parameters which quantitatively and morphologically assessed calcification in the two groups. A non-significant trend in favor of a higher mean and maximal calcified area was detected in patients with T2DM (respectively 0.99 ± 0.72 mm^2^ vs. 0.78 ± 0.49 mm^2^, p = 0.085 and 2.82 ± 2.01 mm^2^ vs. 2.18 ± 1.76 mm^2^, p = 0.085). No differences between patients with and without T2DM were detected in the number of superficial calcifications (superficial-65: 1.93 ± 1.64 vs. 2.06 ± 2.07; superficial-100: 2.37 ± 1.90 vs. 2.65 ± 2.63, all p = ns) or in the percent of superficial calcifications among the total number of deposits (superficial-65: 49.4 ± 34.3% vs. 44.6 ± 33.8%; superficial-100: 59.8 ± 34.7% vs. 57.1 ± 36.1%, all p = ns).

To analyze if the impact of type 2 diabetes on localization, size, shape or extent of calcifications within coronary lesions may be balanced by the potential effect of other clinical and laboratory parameters, covariate adjusting to the propensity score was performed. However and as depicted in Table 2, presence of type 2 diabetes was not associated with significant differences in parameters describing localization, size, shape or extent of calcifications within coronary lesions. Further details are depicted in Table 2.

**Table 2:** OCT-derived analysis of extent and morphology of calcification

	Non DM (49)	DM (56)	p value	p value*
Microcalcifications (present, %)	10 (20.4)	11 (19.6)	NS	NS
Microcalcifications (n per lesion)	0.3 ± 0.7	0.3 ± 0.8	NS	NS
Spotty calcifications (present, %)	46 (93.9)	44 (78.6)	0.025	NS
Spotty calcifications (n per lesion)	2.4 ± 1.9	2.1 ± 1.8	NS	NS
Macrocalcifications (present, %)	34 (69.4)	46 (82.1)	NS	NS
Macrocalcifications (n per lesion)	1.5 ± 0.7	1.5 ± 1.1	NS	NS
Total no. of calcifications (n)	4.2 ± 3.1	4.0 ± 2.6	NS	NS
Superficial65 calcifications (n per lesion)	2.1 ± 2.1	1.9 ± 1.6	NS	NS
Superficial65 calcifications (% of total calcifications)	44.6 ± 33.8	49.4 ± 34.3	NS	NS
Superficial100 calcifications (n per lesion)	2.7 ± 2.6	2.4 ± 1.9	NS	NS
Superficial100 calcifications (% of total calcifications)	57.1 ± 36.1	59.8 ± 34.7	NS	NS
Mean calcium arc (°)	73.7 ± 31.6	82.3 ± 44.8	NS	NS
Mean thickness of calcification (mm)	0.5 ± 0.1	0.5 ± 0.1	NS	NS
Maximal thickness of calcification (mm)	0.9 ± 0.3	1.0 ± 0.3	NS	NS
Mean depth of calcification (µm)	160 ± 76	172 ± 192	NS	NS
Minimal depth of calcification (µm)	62 ± 61	50 ± 71	NS	NS
Mean calcified area (mm^2^)	0.8 ± 0.5	1.0 ± 0.7	0.085	NS
Maximum calcified area (mm^2^)	2.2 ± 1.8	2.8 ± 2.0	0.085	NS
Calcium length (mm)	12.2 ± 9.8	13.6 ± 8.9	NS	NS
Calcium index (° × mm)	1016 ± 958	1311 ± 1312	NS	NS

The data are presented as mean ± SD or n (%). Abbreviations as in Table 1. Superficial65 and Superficial100 calcifications have a minimal depth of 65 and 100 μm, respectively, as defined in the “Methods” section. The data is displayed with p values; p values* are adjusted to the propensity score

### Further characteristics of plaque morphology

**Fig. 3:**
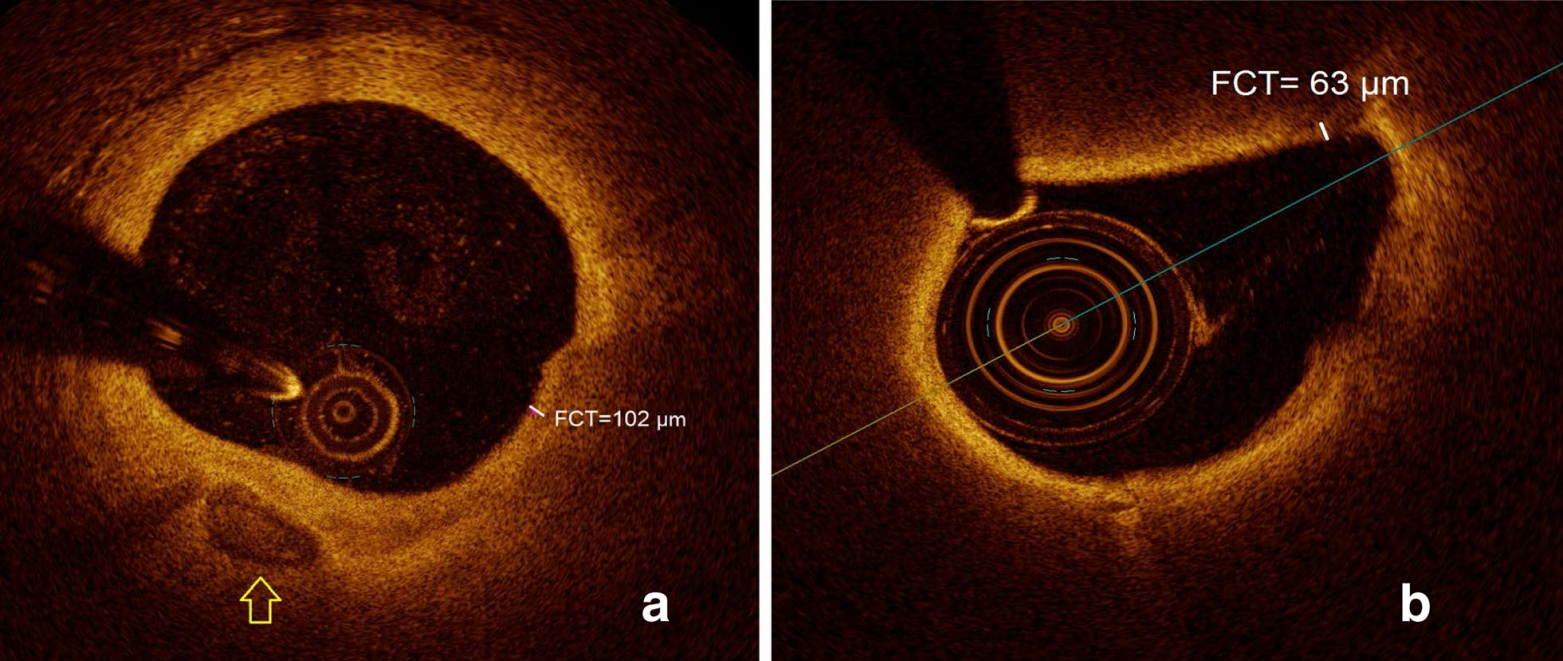
Lipid plaques in patients with (**b**) and without diabetes (**a**). In the presence of diabetes a thinner minimal fibrous cap thickness can be identified. In (**a**) a spotty calcification is visible (marked with the yellow arrow). FCT, fibrous cap thickness

In the patient cohort with T2DM, a lower minimal fibrous cap thickness (FCT) (80.4 ± 27.0 µm vs. 106.8 ± 27.8 µm, p < 0.00001) and a significantly higher percent area stenosis (77.9 ± 8.1% vs. 71.7 ± 11.2%, p < 0.01) was detected, as shown in Fig. 3 and Table 3. Non-significant trends towards a lower mean FCT (125.7 ± 27.0 µm vs. 142.5 ± 27.8 µm, p = 0.07) and a lower mean lipid arc (121.5 ± 40.5° vs. 148.5 ± 50.0°, p = 0.054) were demonstrated in patients with T2DM compared to patients without T2DM. The above described differences in minimal FCT and percent area stenosis remained significant even when co-variates were adjusted to the propensity score (Table 3).

**Table 3:** OCT-derived analysis of further plaque morphology characteristics

	Non DM (49)	DM (56)	p value	p value*
Length of stenosis (mm)	7.7 ± 5.5	8.5 ± 5.7	NS	NS
Percent area stenosis (%)	71.7 ± 11.2	77.9 ± 8.1	0.001	0.019
Minimal FCT (µm)	106.8 ± 27.8	80.4 ± 27.0	< 0.001	0.031
Mean FCT (µm)	142.5 ± 27.8	125.7 ± 27.0	0.070	NS
Mean lipid arc (°)	148.5 ± 50.0	121.5 ± 40.5	0.054	NS

The data are presented as mean ± SD. Abbreviations as in Tables 1 and 2. FCT, fibrous cap thickness. The data is displayed with p values; p values* are adjusted to the propensity score

## Discussion

The main findings of this OCT-investigation are:

- Coronary lesions in patients with T2DM are associated with a lower minimal FCT suggesting a more vulnerable plaque phenotype of the target lesion compared to patients without T2DM.
- There are no significant or relevant differences, neither quantitatively nor qualitatively, regarding localization, size, shape or extent of calcifications in T2DM patients compared to non-diabetics.

Despite recent advances in diagnosis and treatment of atherothrombosis, CAD remains the leading cause of morbidity and mortality in the Western population. Thus, early identification of patients at high cardiovascular risk is necessary. With the emerging use of intracoronary imaging techniques, clinicians have gained more insights into coronary plaque morphology. We and others have previously demonstrated that established features of plaque vulnerability such as a thin fibrous cap overlying the necrotic lipid core, the size of the necrotic lipid core and the presence of macrophages can be visualized in vivo with OCT [6, 26]. These features are often present even in patients with clinically stable CAD [26], thus probably marking a subpopulation at high risk for a future ACS.

It has recently been suggested that localization, size, shape or extent of calcifications within coronary lesions may represent an additional risk factor of coronary plaque vulnerability. Pen et al. [27] demonstrated that patients with a high Framingham risk score have a significantly higher prevalence of coronary calcification compared to those with a low Framingham risk score. Vice versa, the calcium score correlates positively with the incidence of cardiovascular events [7].

With regard to the morphology of calcifications, Vengrenyuk et al. have demonstrated that small superficial calcifications may significantly increase the stress on the fibrous cap; for example, the same fibrous cap stress (300 kPa) in a homogeneous fibrous cap with a thickness of only 65 µm may also be reached in presence of a single, small superficial inclusion in a fibrous cap with a thickness of 200 µm [9]. These data suggest that the inclusion of micro-calcifications within a fibrous cap may mechanically destabilize the fibrous cap and furthermore result in a more vulnerable type of coronary lesion. However, previous studies investigating the localization, size, shape or extent of calcifications used either computed tomography (CT) or IVUS, which are both limited by a lower resolution compared to OCT [28]. Moreover, OCT offers a supreme and established definition of relevant features of vulnerable lipid plaques, such as the minimal FCT, the presence of TCFAs, macrophages, microchannels as well as small calcium deposits [15, 29]. Furthermore, OCT is also effective for the quantification of calcium burden, showing a better correlation with histological assessments compared to IVUS [14]. In addition, OCT is very sensitive to detect superficial calcification and can provide detailed information of calcification morphology, even though it is sometimes difficult to contour calcification shape for deep calcification in certain cases because of the poor penetration of OCT.

As particularly small, superficial calcifications may have an impact on plaque vulnerability, the need for a more precise analysis, exploiting the superior resolution of OCT, has emerged recently. We and others have previously shown that patients with T2DM yield a more vulnerable phenotype of coronary plaques, which may contribute to the higher frequency of cardiovascular events in this population [6, 30, 31]. Part of this vulnerable phenotype may be reverted through statin use, as the OCT-FORMIDABLE register suggests [32], and this effect seems to be enhanced by an optimal glucose control in the diabetic population [33]. The direct, prospective impact of these morphological features on cardiovascular events is currently being analyzed by the ongoing COMBINE study [34]. As micro-calcifications are novel features of coronary plaque vulnerability, our study investigated if the increased cardiovascular risk of patients with T2DM can be explained by an altered localization, size, shape or extent of calcifications within coronary lesions. In the present investigation we extend the current knowledge by demonstrating that patients with T2DM exhibit no significant differences in calcium burden or morphology when compared to patients without T2DM, and these results remained the same following co-variate adjustment to the propensity score. These data are surprising as a recent meta–analysis [30] demonstrated, that in symptomatic patients without known CAD, calcifications assessed through CT scans are more frequent in patients with T2DM. In this study, T2DM seems to be a relevant risk factor for “severe calcification”, but not for mild calcification [30]. Similarly, the MESA Study demonstrated a higher calcium burden in patients with T2DM [35] and Kim et al. pointed out a higher calcium score and a more rapid progression of coronary calcification in patients with metabolic syndrome [36]. However, all of these studies enrolled patients without previously known CAD and performed an image analysis throughout the whole coronary system. Thus, the difference between those investigations and our study may refer to the different study population, study design and imaging modality.

To date only few studies assessed intravascular calcification using the OCT-technique. Krishnamoorty et al. reported a reduced overall calcium volume in the target lesion of patients with T2DM and the authors hypothesized that T2DM might be related to small calcifications with a spotty distribution pattern [37]. On the other hand, a study by Kato et al. [15] showed a higher rate of coronary calcification in patients with T2DM without further detailed and quantitative analysis regarding calcification. In our study, however, we did not observe any difference in localization, size, shape or extent of calcifications using a complex and quantitative frame-by-frame analysis which also assessed the pattern of calcification in detail. In addition, we found a lower minimal FCT and a higher percent area stenosis in patients with T2DM, which is in line with previous studies [15, 31, 38], suggesting a more vulnerable plaque phenotype in patients with T2DM.

Taken together, in the light of our data it is tempting to speculate that the enhanced cardiovascular risk of patients with T2DM cannot be explained by an altered localization, size, shape or extent of calcifications but rather by an altered minimal FCT.

### Limitations

There are several limitations to this study that need to be addressed. Although OCT allows a reliable and effective quantification of calcification [14] and the parameters used to assess localization, size, shape or extent of calcifications are in analogy to the established quantification of the necrotic lipid core, they still need to be validated in a wider population.

Second, due to the potential need of additional contrast medium for the OCT investigation, we excluded patients with chronic kidney disease (CKD) in our study. However, particularly patients with CKD often present with both, increased lesion calcifications and advanced atherosclerosis. Accordingly, we cannot exclude a potential selection bias.

Furthermore, OCT was only performed in the target lesion and 3-vessel OCT was not carried out due to ethical reasons. Thus, comparisons to studies investigating pan-coronary calcification, e.g. using computed tomography is limited.

High-risk characteristics in the diabetic population may be partially influenced by daily glucose fluctuation [39] and may be found already in patients with an impaired glucose tolerance [40]. However, in our study we cannot fully exclude impaired glucose tolerance without overt diabetes mellitus in our non-DM group as oral glucose tolerance testing was not performed routinely.

Although our study is to the best of our knowledge currently the largest study quantifying localization, size, shape or extent of calcifications using a quantitative OCT-analysis and comparing diabetic and non-diabetic patients, it is still relatively small and our findings need to be confirmed in larger populations. Furthermore, our study exclusively included patients with stable angina and we therefore cannot draw any conclusions to patients with ACS—this needs to be assessed in future analyses.

## Conclusion

T2DM has an impact on minimal fibrous cap thickness of coronary target lesion in patients with stable CAD, but not on localization, size, shape or extent of calcifications. Thus, the minimal FCT overlying a necrotic lipid core is likely to be the main factor contributing to the increased plaque vulnerability observed in patients with T2DM.

## Abbreviations

ACS: acute coronary syndrome
CAD: coronary artery disease
CKD: chronic kidney disease
CT: computed tomography
FCT: fibrous cap thickness
FFR: fractional flow reserve
IVUS: intravascular ultrasound
OCT: optical coherence tomography
T2DM: type 2 diabetes mellitus
TCFA: thin-capped fibroatheroma

## References

[1] Kannel WB, McGee DL (1979). Diabetes and cardiovascular disease. The Framingham study. JAMA.

[2] Paneni F, Beckman JA, Creager MA, Cosentino F (2013). Diabetes and vascular disease: pathophysiology, clinical consequences, and medical therapy: part I. Eur Heart J.

[3] Haffner SM, Lehto S, Rönnemaa T, Pyörälä K, Laakso M (1998). Mortality from coronary heart disease in subjects with type 2 diabetes and in nondiabetic subjects with and without prior myocardial infarction. N Engl J Med.

[4] Falk E, Nakano M, Bentzon JF, Finn AV, Virmani R (2013). Update on acute coronary syndromes: the pathologists’ view. Eur Heart J.

[5] Sinclair H, Bourantas C, Bagnall A, Mintz GS, Kunadian V (2015). OCT for the identification of vulnerable plaque in acute coronary syndrome. JACC Cardiovasc Imaging.

[6] Burgmaier M, Hellmich M, Marx N, Reith S (2014). A score to quantify coronary plaque vulnerability in high-risk patients with type 2 diabetes: an optical coherence tomography study. Cardiovasc Diabetol.

[7] Hou ZH, Lu B, Gao Y, Jiang SL, Wang Y, Li W, Budoff MJ (2012). Prognostic value of coronary CT angiography and calcium score for major adverse cardiac events in outpatients. JACC Cardiovasc Imaging.

[8] Kelly-Arnold A, Maldonado N, Laudier D, Aikawa E, Cardoso L, Weinbaum S (2013). Revised microcalcification hypothesis for fibrous cap rupture in human coronary arteries. Proc Natl Acad Sci USA.

[9] Vengrenyuk Y, Carlier S, Xanthos S, Cardoso L, Ganatos P, Virmani R, Einav S, Gilchrist L, Weinbaum S (2006). A hypothesis for vulnerable plaque rupture due to stress-induced debonding around cellular microcalcifications in thin fibrous caps. Proc Natl Acad Sci USA.

[10] Tamaru H, Fujii K, Fukunaga M, Imanaka T, Miki K, Horimatsu T, Nishimura M, Saita T, Sumiyoshi A, Shibuya M (2016). Impact of spotty calcification on long-term prediction of future revascularization: a prospective three-vessel intravascular ultrasound study. Heart Vessels.

[11] Sakaguchi M, Hasegawa T, Ehara S, Matsumoto K, Mizutani K, Iguchi T, Ishii H, Nakagawa M, Shimada K, Yoshiyama M (2016). New insights into spotty calcification and plaque rupture in acute coronary syndrome: an optical coherence tomography study. Heart Vessels.

[12] Jia H, Abtahian F, Aguirre AD, Lee S, Chia S, Lowe H, Kato K, Yonetsu T, Vergallo R, Hu S (2013). In vivo diagnosis of plaque erosion and calcified nodule in patients with acute coronary syndrome by intravascular optical coherence tomography. J Am Coll Cardiol.

[13] Kubo T, Akasaka T, Shite J, Suzuki T, Uemura S, Yu B, Kozuma K, Kitabata H, Shinke T, Habara M (2013). OCT compared with IVUS in a coronary lesion assessment: the OPUS-CLASS study. JACC Cardiovasc Imaging.

[14] Kume T, Okura H, Kawamoto T, Yamada R, Miyamoto Y, Hayashida A, Watanabe N, Neishi Y, Sadahira Y, Akasaka T, Yoshida K (2011). Assessment of the coronary calcification by optical coherence tomography. EuroIntervention.

[15] Kato K, Yonetsu T, Kim SJ, Xing L, Lee H, McNulty I, Yeh RW, Sakhuja R, Zhang S, Uemura S (2012). Comparison of nonculprit coronary plaque characteristics between patients with and without diabetes: a 3-vessel optical coherence tomography study. JACC Cardiovasc Interv.

[16] Burgmaier M, Frick M, Liberman A, Battermann S, Hellmich M, Lehmacher W, Jaskolka A, Marx N, Reith S (2013). Plaque vulnerability of coronary artery lesions is related to left ventricular dilatation as determined by optical coherence tomography and cardiac magnetic resonance imaging in patients with type 2 diabetes. Cardiovasc Diabetol.

[17] Nef H, Elsässer A, Achenbach S, Bergmann M, Brückl R, Byrne R, Gori T, Gutiérrez-Chico JL, Johann K, Krackhardt F, et al. OCT Kompendium. Gießen, Deutschland; 2016.

[18] Reith S, Battermann S, Hoffmann R, Marx N, Burgmaier M (2014). Optical coherence tomography derived differences of plaque characteristics in coronary culprit lesions between type 2 diabetic patients with and without acute coronary syndrome. Catheter Cardiovasc Interv.

[19] Reith S, Battermann S, Jaskolka A, Lehmacher W, Hoffmann R, Marx N, Burgmaier M (2013). Predictors and incidence of stent edge dissections in patients with type 2 diabetes as determined by optical coherence tomography. Int J Cardiovasc Imaging.

[20] Reith S, Battermann S, Jaskolka A, Lehmacher W, Hoffmann R, Marx N, Burgmaier M (2013). Relationship between optical coherence tomography derived intraluminal and intramural criteria and haemodynamic relevance as determined by fractional flow reserve in intermediate coronary stenoses of patients with type 2 diabetes. Heart.

[21] Tearney GJ, Regar E, Akasaka T, Adriaenssens T, Barlis P, Bezerra HG, Bouma B, Bruining N, Cho JM, Chowdhary S (2012). Consensus standards for acquisition, measurement, and reporting of intravascular optical coherence tomography studies: a report from the International Working Group for Intravascular Optical Coherence Tomography Standardization and Validation. J Am Coll Cardiol.

[22] Ong DS, Lee JS, Soeda T, Higuma T, Minami Y, Wang Z, Lee H, Yokoyama H, Yokota T, Okumura K, Jang IK (2016). Coronary calcification and plaque vulnerability: an optical coherence tomographic study. Circ Cardiovasc Imaging..

[23] Mizukoshi M, Kubo T, Takarada S, Kitabata H, Ino Y, Tanimoto T, Komukai K, Tanaka A, Imanishi T, Akasaka T (2013). Coronary superficial and spotty calcium deposits in culprit coronary lesions of acute coronary syndrome as determined by optical coherence tomography. Am J Cardiol.

[24] Ehara S, Kobayashi Y, Yoshiyama M, Shimada K, Shimada Y, Fukuda D, Nakamura Y, Yamashita H, Yamagishi H, Takeuchi K (2004). Spotty calcification typifies the culprit plaque in patients with acute myocardial infarction: an intravascular ultrasound study. Circulation.

[25] Kataoka Y, Puri R, Hammadah M, Duggal B, Uno K, Kapadia SR, Tuzcu EM, Nissen SE, Nicholls SJ (2014). Spotty calcification and plaque vulnerability in vivo: frequency-domain optical coherence tomography analysis. Cardiovasc Diagn Ther.

[26] Iannaccone M, Quadri G, Taha S, D’Ascenzo F, Montefusco A, Omede’ P, Jang IK, Niccoli G, Souteyrand G, Yundai C (2016). Prevalence and predictors of culprit plaque rupture at OCT in patients with coronary artery disease: a meta-analysis. Eur Heart J Cardiovasc Imaging.

[27] Pen A, Yam Y, Chen L, Dennie C, McPherson R, Chow BJ (2013). Discordance between Framingham Risk Score and atherosclerotic plaque burden. Eur Heart J.

[28] van der Giessen AG, Gijsen FJ, Wentzel JJ, Jairam PM, van Walsum T, Neefjes LA, Mollet NR, Niessen WJ, van de Vosse FN, de Feyter PJ, van der Steen AF (2011). Small coronary calcifications are not detectable by 64-slice contrast enhanced computed tomography. Int J Cardiovasc Imaging.

[29] Nasu K, Tsuchikane E, Katoh O, Fujita H, Surmely JF, Ehara M, Kinoshita Y, Tanaka N, Matsubara T, Asakura Y (2008). Plaque characterisation by Virtual Histology intravascular ultrasound analysis in patients with type 2 diabetes. Heart.

[30] Nicoll R, Zhao Y, Ibrahimi P, Olivecrona G, Henein M (2016). Diabetes and hypertension consistently predict the presence and extent of coronary artery calcification in symptomatic patients: a systematic review and meta-analysis. Int J Mol Sci.

[31] De Rosa R, Vasa-Nicotera M, Leistner DM, Reis SM, Thome CE, Boeckel JN, Fichtlscherer S, Zeiher AM (2017). Coronary atherosclerotic plaque characteristics and cardiovascular risk factors—insights from an optical coherence tomography study. Circ J.

[32] Gili S, Iannaccone M, Colombo F, Montefusco A, Amabile N, Calcagno S, Capodanno D, Scalone G, Rognoni A, Omedè P, et al. Effects of statins on plaque rupture assessed by optical coherence tomography in patients presenting with acute coronary syndromes: insights from the optical coherence tomography (OCT)-FORMIDABLE registry. Eur Heart J Cardiovasc Imaging. 2017. 10.1093/ehjci/jex102. 10.1093/ehjci/jex102 28605473

[33] Dong N, Xie Z, Dai J, Wang W, Sun R, Zhan Y, Sun M, Tian J, Yu B (2016). Statin-induced improvements in vulnerable plaques are attenuated in poorly controlled diabetic patients with coronary atherosclerosis disease: a serial optical coherence tomography analysis. Acta Diabetol.

[34] Kennedy MW, Fabris E, Ijsselmuiden AJ, Nef H, Reith S, Escaned J, Alfonso F, van Royen N, Wojakowski W, Witkowski A (2016). Combined optical coherence tomography morphologic and fractional flow reserve hemodynamic assessment of non-culprit lesions to better predict adverse event outcomes in diabetes mellitus patients: COMBINE (OCT-FFR) prospective study. Rationale and design. Cardiovasc Diabetol.

[35] Wong ND, Nelson JC, Granston T, Bertoni AG, Blumenthal RS, Carr JJ, Guerci A, Jacobs DR, Kronmal R, Liu K (2012). Metabolic syndrome, diabetes, and incidence and progression of coronary calcium: the Multiethnic Study of Atherosclerosis study. JACC Cardiovasc Imaging.

[36] Kim LK, Yoon JW, Lee DH, Kim KM, Choi SH, Park KS, Jang HC, Kim MK, Park HE, Choi SY, Lim S (2016). Impact of metabolic syndrome on the progression of coronary calcium and of coronary artery disease assessed by repeated cardiac computed tomography scans. Cardiovasc Diabetol.

[37] Krishnamoorthy P, Vengrenyuk Y, Ueda H, Yoshimura T, Pena J, Motoyama S, Baber U, Hasan C, Kesanakurthy S, Sweeny JM (2017). Three-dimensional volumetric assessment of coronary artery calcification in patients with stable coronary artery disease by OCT. EuroIntervention.

[38] Fukunaga M, Fujii K, Nakata T, Shibuya M, Miki K, Kawasaki D, Masutani M, Kawabata-Lee M, Ohyanagi M, Masuyama T (2012). Multiple complex coronary atherosclerosis in diabetic patients with acute myocardial infarction: a three-vessel optical coherence tomography study. EuroIntervention.

[39] Kuroda M, Shinke T, Sakaguchi K, Otake H, Takaya T, Hirota Y, Osue T, Kinutani H, Konishi A, Takahashi H (2015). Association between daily glucose fluctuation and coronary plaque properties in patients receiving adequate lipid-lowering therapy assessed by continuous glucose monitoring and optical coherence tomography. Cardiovasc Diabetol.

[40] Suzuki K, Takano H, Kubota Y, Inui K, Nakamura S, Tokita Y, Kato K, Asai K, Shimizu W (2016). Plaque characteristics in coronary artery disease patients with impaired glucose tolerance. PLoS ONE.

